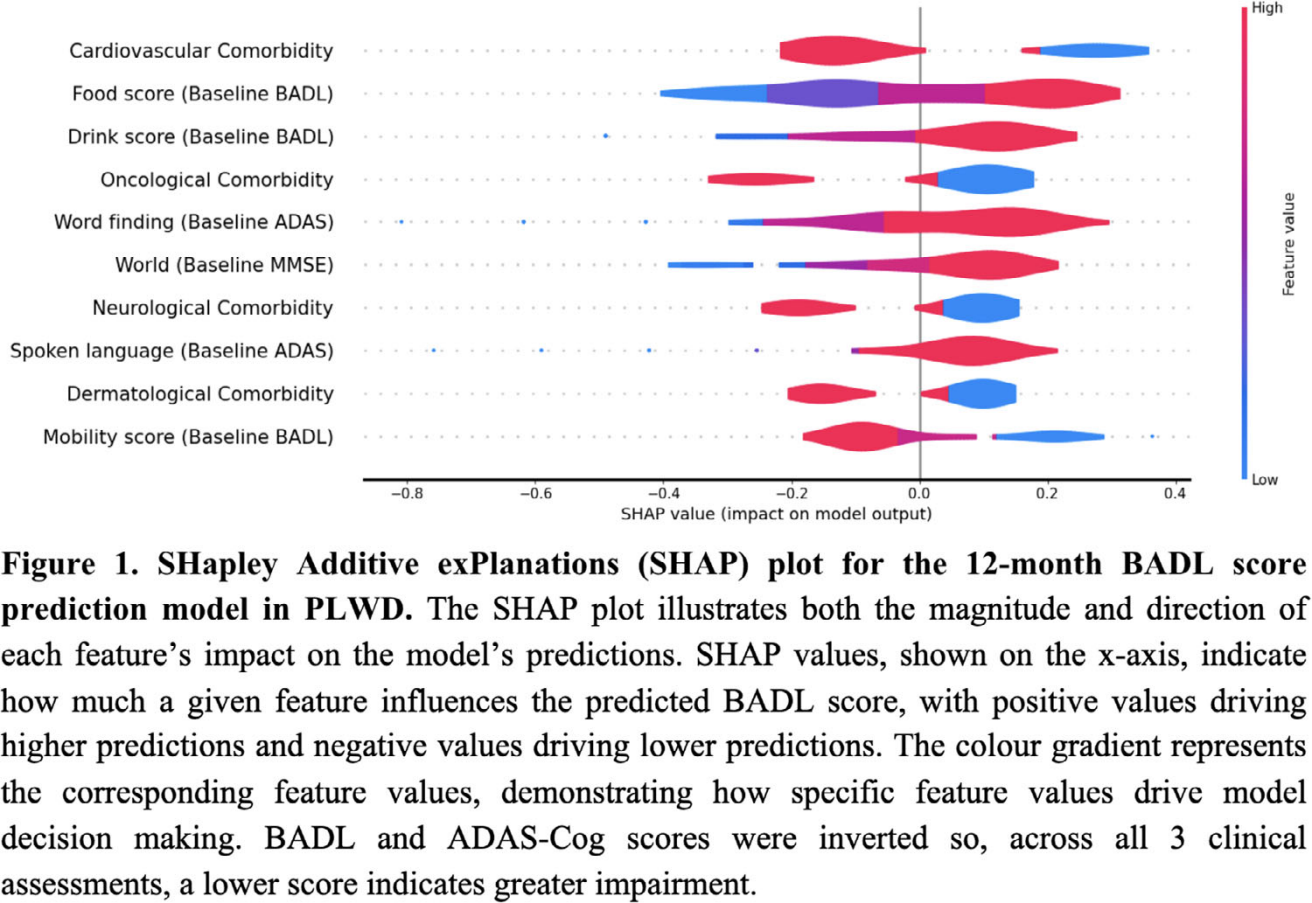# A digital consultation tool for predicting rate of decline in activities of daily living in people living with dementia

**DOI:** 10.1002/alz70858_102593

**Published:** 2025-12-25

**Authors:** Antigone Fogel, Chloe Walsh, Nan Fletcher‐Lloyd, Mina Ryten, Paresh Malhotra, Ramin Nilforooshan, Payam Barnaghi, CR&T Group

**Affiliations:** ^1^ UK Dementia Research Institute, Care Research and Technology Centre, London, United Kingdom; ^2^ Imperial College London, London, United Kingdom; ^3^ UK Dementia Research Institute, Care Research and Technology Centre, Imperial College London, London, United Kingdom; ^4^ Surrey and Borders Partnership NHS Foundation Trust, Leatherhead, United Kingdom; ^5^ Cambridge University, Cambridge, United Kingdom; ^6^ UK Dementia Research Institute, Cambridge, United Kingdom; ^7^ Imperial College Healthcare NHS Trust, London, United Kingdom

## Abstract

**Background:**

As the population of people living with dementia (PLWD) increases, the need for scalable, high‐quality, and personalised patient care will increase as well. A key consideration during dementia care planning is a patient's ability to perform activities of daily living (ADL), as this not only determines the level of care they require, but also directly impacts their independence and overall quality of life. However, despite considerable variability in trajectories of functional decline across PLWD, clinicians currently rely on experience and generalised statistics to inform care decisions. Thus, there is a need for more tailored approaches that predict how each patient's functional abilities will change over time. A data‐driven decision support tool could address this gap by anticipating care needs, improving resource allocation, and supporting patient autonomy and quality of life.

**Method:**

Longitudinal, observational data from 118 PLWD (761 assessments total) including clinical assessment, demographic, and medical history information was used. Clinical assessments included the Mini‐Mental State Exam (MMSE), Alzheimer's Disease Assessment Scale‐Cognitive Subscale (ADAS‐Cog), and Bristol Activities of Daily Living questionnaire (BADL), and comorbidities were classified according to ICD‐10 chapters. A regression model was developed to predict functional decline over a 12‐month period, as measured by BADL, in PLWD. To support explainability and use of this model in clinical settings, a digital consultation tool was developed.

**Result:**

On average, participants declined at a rate of 4.2 (SD 5.7) BADL score‐points per year. The best‐performing machine learning model was a ridge regression model that predicted BADL scores 12 months in the future with a mean absolute error (MAE) of 4.0 (SD 0.4). Ability to prepare food and drink, presence of a cardiovascular or oncological comorbidity, and language abilities at baseline were found to be most predictive of 12‐month functional decline in PLWD.

**Conclusion:**

Our model, developed using readily available clinical features, is well‐suited for implementation into UK memory clinics. A digital consultation tool was designed to enhance their clinical utility. Given the critical importance of predicting a person's future functional ability during care planning, this tool has the potential to augment care pathways by providing personalised prognostic insights into future care needs.